# Unemployment and cause-specific mortality among the Belgian working-age population: The role of social context and gender

**DOI:** 10.1371/journal.pone.0216145

**Published:** 2019-05-02

**Authors:** Katrien Vanthomme, Sylvie Gadeyne

**Affiliations:** Interface Demography, Department of Social Research, Faculty of Economic and Social Sciences & Solvay Business School, Vrije Universiteit Brussel, Brussels, Belgium; Sciensano, BELGIUM

## Abstract

**Background:**

Life expectancy increased in industrialized countries, but inequalities in health and mortality by socioeconomic position (SEP) still persist. Several studies have documented educational inequalities, yet the association between health and employment status remains unclear. However, this is an important issue considering the instability of the labour market and the fact that unemployment now also touches ‘non-traditional groups’ (e.g. the high-educated). This study will 1) probe into the association between unemployment and cause-specific mortality; 2) look into the possible protective effect of sociodemographic variables; 3) assess the association between unemployment, SEP, gender and cause-specific mortality.

**Material and methods:**

Individually linked data of the Belgian census (2001) and Register data on emigration and cause-specific mortality during 2001–2011 are used. The study population contains the Belgian population eligible for employment at census, based on age (25–59 years) and being in good health. Both absolute and relative measures of all-cause and cause-specific mortality by employment status have been calculated, stratified by gender and adjusted for sociodemographic and socioeconomic indicators.

**Results:**

Unemployed men and women were at a higher risk for all-cause and cause-specific mortality compared with their employed counterparts. The excess mortality among unemployed Belgians was particularly high for endocrine and digestive diseases, mental disorders, and falls, and more pronounced among men than among women. Other indicators of SEP did only slightly decrease the mortality disadvantage of being unemployed.

**Discussion:**

The findings stress the need for actions to ameliorate the health status of unemployed people, especially for the most vulnerable groups in society.

## Introduction

Life expectancy has increased substantially in industrialized countries in the last decades [1]. However, important differences in life expectancy, morbidity and mortality are observed between socioeconomic (SE) groups. Reducing SE inequalities in health and mortality are one of the major challenges of public health policies nowadays, even in western countries with high-quality and accessible health care systems [2,3]. In some settings, SE inequalities are even increasing, mostly related to SE disparities in unhealthy behaviours [4]. While many studies have documented educational inequalities in morbidity and mortality, employment status has less often been used as indicator of socioeconomic position (SEP) in relation to health.

This study aims to fill this gap by analysing inequalities in mortality by employment status. This is an important issue to address firstly because the likelihood of getting unemployed at a certain point in time has increased considerably due to the instability of the labour market; and secondly because unemployment now also touches ‘non-traditional groups’, such as the higher educated and the higher socio-professional groups [5]. The association between unemployment and mortality however may be different depending on the other social strata people belong to [6]. Therefore, we believe that investigating the association between unemployment and cause-specific mortality, accounting for other measures of social disadvantage is worthwhile.

As a result of the shifts in the global economic market and the economic recession, employment patterns have changed from stable and predictable to more flexible and uncertain [7]. Consequently, the probability of getting unemployed has increased in recent decades, which could possibly have a rather large public health impact [1,7–9]. Indeed, many studies have suggested that being unemployed can be harmful in several ways, not only financially and psychosocially, but also healthwise [1,7,10–17]. Unemployment has shown to be associated with both mental and physical ill-health and mortality [7,8,10–12,15–19,20–22]. Yet, there might be a protective effect of contextual economic recession in which mortality rates tend to fall [8,15,16,21,23]. The association is even observed in countries with established social welfare systems, which are set up to protect against the negative effects from unemployment [7,8].

As mentioned earlier, unemployment is now also increasingly affecting other groups than the ‘traditional ones’ [9], e.g. the young, the high-educated, or women. Yet, few studies have paid attention as to whether the association between unemployment and health varies by other indicators of social disadvantage. Such studies could however gain information on the pathways linking unemployment and health.

The literature on the link between unemployment and health is quite controversial, with a lot of debate on the causal direction of this relation [9,14–17]. Some authors claim that unemployment is associated with poor health because of selection processes [17,22]. Yet, health selection in unemployment is likely to be weaker in times when unemployment levels are high [9,15–17]. Moreover, adverse health effects have been observed for the relatives of the unemployed as well, thereby excluding a health selection effect [10,20]. Contrariwise, the causal relation from unemployment to poor health might be explained by psychosocial resources and stress, limited material and financial means (hence poor food habits or reductions in health care expenditure), or adverse health behaviours such as tobacco or alcohol consumption [12,13,17,19,20,22,24–26]. These detrimental effects of being unemployed may vary by the individual social environment [9,13] such as the family context and other sociodemographic indicators.

The aim of this study is threefold: first, we want to assess whether unemployment is associated with higher mortality risks and if so for which specific causes of death (COD). Next, as single indicators of SEP do not maximally discriminate between social groups [27], we will add other socioeconomic (i.e. educational attainment and home ownership) and sociodemographic indicators (i.e. the presence of a partner and migrant background) into the models to see whether these may protect against the detrimental health effects of being unemployed. Finally, we want to assess whether the association between unemployment, SEP and cause-specific mortality varies by gender.

## Material and methods

### Design and study population

Analyses were based on a population-wide dataset, consisting of a record linkage between the 2001 Belgian census and Register data on emigration and cause-specific mortality for the period 1^st^ October 2001-31^st^ December 2011. The dataset contained census-based information on socio-demographic and SE characteristics as well as cause-specific mortality figures for the total de jure Belgian population.

The study population comprised the Belgian population aged 25 to 59 years at census 2001, eligible for employment and in good health (N = 3,084,137). Limiting the analyses to the 25 to 59-year olds excludes those who may be still studying (<25 years) and those who are at the end-of-career periods (60 and older), which leads to a more homogenous study population. Moreover, to capture the population eligible for employment, students, retirees and people not working because of personal, social or health reasons as well as unemployed people not actively looking for a job were excluded from the study population. To exclude a health selection effect, people that claimed to be in ‘fair’, ‘poor’ or ‘very poor’ health at the moment of the 2001 census were also excluded from the analyses.

### Variables

The study population was classified by employment status at the time of the census 2001, contrasting employed and unemployed people who were actively looking for a job. Differences in cause-specific mortality by employment status were controlled for the following social characteristics: educational attainment, home ownership, living situation, and migration background. Educational attainment was categorized according to the International Standard Classification of Education [28]: lower secondary education or less (ISCED 0–2, “low”), upper secondary education (ISCED 3–4, “mid”), and tertiary education (ISCED 5–6, “high”). Home ownership differentiates between tenants and owners of a dwelling. Living situation discriminates people living without partner from people living with partner. Migration background separates native Belgians from non-native Belgians.

COD were classified according to the International Classification of Diseases and Health-related problems, tenth revision (ICD-10). The COD were grouped according to the chapters of ICD-10 (highlighted in grey in the cause-specific tables and called ‘major groups of COD’)). In addition, major single COD such as diabetes, ischaemic heart disease and transport accidents were examined separately. Table 1 gives an overview of the COD, the ICD-10 codes and the number of deaths for Belgian men and women.

**Table 1:** Causes of death, corresponding ICD-10 codes and number of deaths by gender during 2001–2011 in the Belgian study population aged 25–59 years.

	ICD-10	Male deaths	Female deaths
All deaths	A00-Y98	43,834	16,348
Infectious diseases	A00–B99	568	208
Cancers	C00–D48	16,162	8,863
Endocrine diseases	E00–E90	490	134
Diabetes	E10-14	259	57
Mental and behavioural disorders	F00–F99	670	170
Mental disorder due to alcohol	F10	498	113
Diseases of the nervous system	G00–G99	707	271
Diseases of the circulatory system	I00–I99	8,757	2,080
Hypertensive diseases	I10-15	158	43
Ischaemic Heart Disease	I20-25	4,263	556
Pulmonary Heart Disease	I26-28	287	112
Diseases of the respiratory system	J00–J99	1,165	336
Pneumonia	J12-18	318	91
Chronic lower respiratory infections	J40-47	522	164
Diseases of the digestive system	K00–K93	2,348	769
Alcoholic liver disease	K70	1,111	376
Fibrosis and cirrhosis of the liver	K74	471	150
Injury, poisoning and certain other consequences of external causes	S00–T98	5,968	1,636
External causes of morbidity and mortality	V01–Y98	3,956	975
Transport accidents	V01-99	1,089	240
Falls	W00-19	293	80
Intentional self-harm	X60-84	1,891	462

### Statistical analyses

To get the full picture of mortality inequalities by employment status, both absolute and relative measures were calculated, as is recommended [29–33]. First, for all-cause and cause-specific mortality, directly age-standardized mortality rates (ASMR) were calculated by employment status and gender, using the total Belgian population at the 2001 census as standard population. We then calculated the mortality rate difference (MRD), which is the result of the difference between the ASMR of the unemployed and the ASMR of the employed group. Secondly, all-cause and cause-specific mortality rate ratios (MRR) were calculated using Poisson regression models. In a first step, these models were only adjusted for attained age; in a second step, the other indicators of social disadvantage were added to the model. Furthermore, for all-cause mortality the association between the cross-classifications of employment status and the other measures of social disadvantage was assessed using both ASMR and MRR. Additionally, we performed some sensitivity analysis among the ‘unhealthy group’. We assessed whether the association between employment status and all-cause and cause-specific mortality holds true among the unhealthy group as well. To study this, both ASMR and MRR were calculated for all-cause and cause-specific mortality. The results are available upon request and are shortly discussed in the discussion section. All analyses were performed using Stata/MP 14.2.

## Results

### Description of the study population

In 2001, five percent of the Belgian men belonging to the population eligible to be at work was unemployed and looking for a job, while for women, this percentage was twice as high (Table 2).

**Table 2:** Description of the study population at census 2001 by variables of interest—Belgian men and women aged 25–59 years.

		Employed		Unemployed and looking for a job	
		Number	%	Number	%
Men		1,616,949	95.46	76,850	4.54
Educational attainment	Low	494,004	31.14	35,283	48.12
	Mid	559,808	35.29	23,462	32.00
	High	532,471	33.57	14,579	19.88
Home ownership	Owner	1,227,755	77.89	35,222	48.59
	Tenant	348,612	22.11	37,268	51.41
Living situation	With partner	1,197,502	75.54	33,498	45.41
	Without partner	387,770	24.46	40,266	54.59
Migrant background	Native	1,436,837	88.86	53,893	70.13
	Non-native	180,112	11.14	22,957	29.87
Women		1,259,780	90.61	130,558	9.39
Educational attainment	Low	288,115	23.16	56,333	44.26
	Mid	424,166	34.10	48,654	38.23
	High	531,595	42.74	22,285	17.51
Home ownership	Owner	945,982	77.02	74,468	59.26
	Tenant	282,190	22.98	51,192	40.74
Living situation	With partner	936,518	75.48	79,685	62.10
	Without partner	304,171	24.52	48,630	37.90
Migrant background	Native	1,139,717	90.47	100,624	77.07
	Non-native	120,063	9.53	29,934	10.79

Compared with employed men and women, the unemployed were more likely to be in a socially disadvantaged position. While women were more often higher educated than men, both unemployed men and women were more often lower educated compared with their employed counterparts. Almost three out of four employed men and women were owner of a dwelling, but this percentage decreased among the unemployed to about 50% of the unemployed men and 60% of the unemployed women. Among men, the unemployed were more likely to be living without partner, whereas the majority of the women was living with a partner even if the proportion of women living without partner was higher among the unemployed. Finally, the percentage of men and women with a migrant background was higher among the unemployed than among the employed.

### Is being unemployed associated with higher all-cause and cause-specific mortality within the Belgian working age population?

Unemployment was associated with higher all-cause and cause-specific mortality (Tables 3 and 4). This association was stronger among men than among women: in absolute terms employed men had an all-cause ASMR of 243.1/100,000 person-years (95% CI: 240.8–245.5) whereas unemployed men had an all-cause ASMR of 565.8/100,000 person-years (95% CI: 548.3–583.4) resulting in a mortality rate difference of 322.7/100,000 (Table 3). Among women, the absolute difference in all-cause mortality between unemployed and employed women was much smaller: 73.2/100,000 (ASMR_unemployed_ = 195.1/100,000 –ASMR_employed_ = 121.9/100,000). In relative terms, male all-cause mortality was twice as high among the unemployed compared with the employed (MRR: 2.32; 2.24–2.40), while among unemployed women, all-cause mortality was 64% higher compared with employed women (MRR: 1.64; 1.57–1.72) (Table 4).

**Table 3:** Age-standardized all-cause and cause-specific mortality rates (ASMR) per 100,000 person-years with 95% confidence intervals (CI) by employment status and gender, 2001–2011, Belgian men and women aged 25–59 years.

	Men			Women		
	Employed	Unemployed and looking for a job	MRD	Employed	Unemployed and looking for a job	MRD
All deaths	243.1 (240.8–245.5)	565.8 (548.3–583.4)	322.7	121.9 (119.9–123.9)	195.1 (187.1–203.1)	73.2
Infectious diseases	3.1 (2.8–3.3)	10.4 (8.0–12.8)	7.3	1.6 (1.3–1.8)	3.2 (2.1–4.2)	1.6
Cancers	91.0 (89.6–92.4)	170.7 (161.0–180.3)	79.7	69.1 (67.6–70.6)	88.1 (82.7–93.5)	19.0
Endocrine diseases	2.6 (2.3–2.8)	9.5 (7.2–11.8)	6.9	0.9 (0.8–1.1)	2.6 (1.6–3.5)	1.7
Diabetes	1.4 (1.2–1.6)	4.3 (2.8–5.9)	2.9	0.4 (0.3–0.5)	1.4 (0.7–2.1)	1.0
Mental disorders	3.2 (2.9–3.4)	21.5 (18.0–25.0)	18.3	1.1 (0.9–1.3)	3.9 (2.7–5.0)	2.8
Mental disorder due to alcohol	2.3 (2.1–2.5)	17.2 (14.1–20.4)	14.9	0.7 (0.5–0.8)	2.8 (1.8–3.7)	2.1
Diseases of the nervous system	3.9 (3.6–4.2)	9.9 (7.5–12.2)	6.0	2.2 (1.9–2.5)	2.6 (1.6–3.5)	0.4
Diseases of the circulatory system	49.0 (48.0–50.0)	107.0 (99.3–114.6)	58.0	15.7 (15.0–16.4)	27.4 (24.4–30.4)	11.7
Hypertensive diseases	0.9 (0.7–1.0)	1.5 (0.6–2.4)	0.6	0.3 (0.2–0.5)	0.7 (0.2–1.2)	0.4
Ischaemic Heart Disease	23.9 (23.2–24.6)	47.2 (42.1–52.3)	23.3	4.3 (3.9–4.7)	7.3 (5.8–8.9)	3.0
Pulmonary Heart Disease	1.6 (1.4–1.8)	3.3 (2.0–4.7)	1.7	0.7 (0.6–0.9)	1.9 (1.1–2.6)	1.2
Diseases of the respiratory system	6.3 (6.0–6.7)	19.4 (16.1–22.6)	13.1	2.4 (2.1–2.7)	6.5 (5.0–7.9)	4.1
Pneumonia	1.7 (1.5–1.9)	5.4 (3.7–7.2)	3.7	0.6 (0.5–0.8)	1.7 (1.0–2.5)	1.1
Chronic lower respiratory infections	2.8 (2.6–3.0)	9.3 (7.1–11.6)	6.5	1.2 (1.0–1.4)	3.6 (2.5–4.7)	2.4
Diseases of the digestive system	12.2 (11.7–12.7)	47.2 (42.0–52.4)	35.0	4.9 (4.5–5.3)	15.2 (13.0–17.4)	10.3
Alcoholic liver disease	5.6 (5.2–6.0)	24.3 (20.6–28.0)	18.7	2.3 (2.1–2.6)	7.9 (6.3–9.5)	5.6
Fibrosis and cirrhosis of the liver	2.4 (2.2–2.7)	9.1 (6.8–11.4)	6.7	0.9 (0.8–1.1)	3.0 (2.0–4.0)	2.1
Injury, poisoning and other causes	34.2 (33.3-.35.1)	54.5 (49.1–59.9)	20.3	11.2 (10.6–11.8)	17.6 (15.3–20.0)	6.4
External causes of morbidity and mortality	21.3 (20.6–22.0)	64.6 (58.7–70.4)	43.3	6.2 (5.7–6.6)	15.2 (13.1–17.4)	9.0
Transport accidents	6.1 (5.7–6.5)	14.2 (11.5–16.9)	8.1	1.6 (1.4–1.8)	3.1 (2.2–4.1)	1.5
Falls	1.5 (1.3–1.7)	6.9 (5.0–8.9)	5.4	0.5 (0.4–0.7)	1.7 (1.0–2.5)	1.2
Intentional self-harm	10.3 (9.8–10.7)	27.7 (23.9–31.4)	17.4	2.9 (2.6–3.2)	6.9 (5.5–8.4)	4.0

Number of men = 1,693,799: 1,616,949 employed and 76,850 unemployed and looking for a job. Number of women = 1,390,338: 1,259,780 employed and 130,558 unemployed and looking for a job. MRD = Mortality Rate Difference ASMR_unemployed_−ASMR_employed_

**Table 4:** Age-adjusted all-cause and cause-specific mortality rate ratios (MRR) with 95% confidence intervals (CI) of being unemployed but looking for a job versus being unemployed, with and without adjustment for educational attainment, 2001–2011, Belgian men and women aged 25–59 years.

	Men		Women	
	Model 1	Model 2	Model 1	Model 2
*Reference category is employed*	MRR (95% CI)	MRR (95% CI)	MRR (95% CI)	MRR (95% CI)
All deaths	2.32 (2.24–2.40)	1.80 (1.74–1.87)	1.64 (1.57–1.72)	1.48 (1.41–1.56)
Infectious diseases	3.50 (2.73–4.50)	2.52 (1.89–3.35)	2.10 (1.46–3.01)	1.77 (1.21–2.59)
Cancers	1.86 (1.75–1.98)	1.55 (1.45–1.66)	1.30 (1.21–1.39)	1.21 (1.14–1.31)
Endocrine diseases	3.60 (2.75–4.70)	2.56 (1.89–3.46)	2.77 (1.83–4.19)	2.44 (1.57–3.80)
Diabetes	3.12 (2.12–4.59)	2.07 (1.35–3.18)	3.56 (1.96–6.45)	3.07 (1.64–5.74)
Mental disorders	6.90 (5.74–8.29)	3.80 (3.06–4.73)	3.53 (2.50–4.99)	2.70 (1.84–3.96)
Mental disorder due to alcohol	7.60 (6.17–9.37)	4.11 (3.19–5.29)	4.26 (2.84–6.38)	3.22 (2.10–4.95)
Diseases of the nervous system	2.59 (2.02–3.33)	2.00 (1.50–2.67)	1.14 (0.77–1.68)	1.17 (0.77–1.78)
Diseases of the circulatory system	2.20 (2.03–2.37)	1.70 (1.56–1.85)	1.78 (1.58–2.02)	1.52 (1.33–1.73)
Hypertensive diseases	1.86 (1.01–3.43)	1.55 (0.82–2.93)	2.37 (1.10–5.12)	1.91 (0.86–4.24)
Ischaemic Heart Disease	1.99 (1.77–2.24)	1.60 (1.41–1.82)	1.81 (1.43–2.28)	1.55 (1.21–1.99)
Pulmonary Heart Disease	2.09 (1.35–3.24)	1.84 (1.13–3.00)	2.47 (1.54–3.95)	2.42 (1.46–4.00)
Diseases of the respiratory system	3.14 (2.62–3.76)	2.10 (1.70–2.59)	2.75 (2.11–3.57)	2.23 (1.68–2.94)
Pneumonia	3.44 (2.46–4.81)	2.53 (1.72–3.73)	3.15 (1.96–5.08)	2.44 (1.46–4.08)
Chronic lower respiratory infections	3.43 (2.64–4.45)	2.22 (1.63–3.02)	3.13 (2.18–4.51)	2.41 (1.63–3.56)
Diseases of the digestive system	3.83 (3.40–4.32)	2.74 (2.39–3.15)	3.15 (2.66–3.72)	2.71 (2.26–3.25)
Alcoholic liver disease	4.20 (3.54–4.97)	3.18 (2.62–3.86)	3.44 (2.72–4.35)	3.10 (2.41–4.00)
Fibrosis and cirrhosis of the liver	3.78 (2.89–4.95)	2.93 (2.15–3.98)	3.20 (2.19–4.69)	2.70 (1.80–4.06)
Injury, poisoning and other consequences of external causes	1.55 (1.40–1.71)	1.28 (1.14–1.43)	1.58 (1.37–1.82)	1.45 (1.24–1.69)
External causes of morbidity and mortality	3.06 (2.78–3.37)	2.36 (2.11–2.63)	2.64 (2.26–3.08)	2.13 (1.80–2.53)
Transport accidents	2.29 (1.88–2.79)	1.79 (1.43–2.23)	2.13 (1.54–2.94)	1.60 (1.10–2.33)
Falls	4.77 (3.50–6.49)	3.12 (2.20–4.43)	3.68 (2.24–6.06)	2.70 (1.52–4.77)
Intentional self-harm	2.83 (2.46–3.27)	2.35 (2.00–2.76)	2.61 (2.09–3.27)	2.30 (1.80–2.94)

Mortality rate ratio of being unemployed and looking for a job versus being employed. Model 1: baseline model adjusted for attained age; Model 2: baseline model adjusted for attained age, educational attainment, home ownership, living situation and migrant background. Number of men = 1,693,799; number of women: 1,390,338.

This adverse association between unemployment and mortality (measured both absolute and relative) was persistent for all major groups of COD, as well as for all major single COD, both among men and women. The only exceptions were diseases of the nervous system among women (absolute and relative inequalities) and hypertensive diseases among men and women (absolute inequalities only). For cause-specific mortality also, the association between unemployment and mortality was larger among men than among women.

Among the major groups of COD (highlighted in grey), in absolute terms, the largest inequalities between employed and unemployed people were found for cancers, diseases of the circulatory system and external causes (only men), as these are also the largest COD (Table 3). The largest relative mortality inequalities between employed and unemployed men and women were observed for deaths due to mental disorders with MRRs of respectively 6.90 (95% CI: 5.74–8.29) for men and 3.53 (95% CI: 2.50–4.99) for women (Table 4).

For the other major groups of COD, there also was a large excess mortality among unemployed men and women. For digestive, endocrine, infectious and respiratory diseases as well as external COD, mortality among the unemployed was more than three times as high among men and more than two times higher among women, compared with the employed.

To be more specific by taking a look at the major single COD, excess mortality among unemployed men and women was observed for all these COD. The largest relative mortality inequality between employed and unemployed men and women was noted for mortality due to mental disorders related to alcohol, resulting in MRRs of respectively 7.60 (95% CI: 6.17–9.37) for men and 4.26 (95% CI: 2.84–6.38) for women (Table 4). The excess mortality due to falls was also very large among the unemployed with mortality rates that were almost five times as high among men (4.77; 95% CI: 3.50–6.49) and almost four times as high among women (3.68; 95% CI: 2.24–6.06). Large mortality inequalities were also observed for respiratory and digestive diseases: relative mortality inequalities for alcoholic liver disease, liver cirrhosis, pneumonia and chronic lower respiratory infections were among the largest for both men and women with MRRs above three. In addition, unemployed men and women had a three times higher chance of dying from diabetes (MRR_men_: 3.12; 95% CI: 2.12–4.59 and MRR_women_: 3.56; 95% CI: 1.96–6.45) compared with employed men and women.

### Do other measures of social disadvantage alter the association between unemployment and all-cause and cause-specific mortality?

Table 5 shows the results of the absolute and relative cross-classification analyses for all-cause mortality. Looking at the cross-classification of education and unemployment, we observed that unemployment discriminates more in terms of mortality than education, showing higher mortality among the unemployed, irrespective of their educational level, especially for men.

**Table 5:** Age-standardized all-cause mortality rates (ASMR) per 100,000 person-years and age-adjusted all-cause mortality rate ratios (MRR) with 95% confidence intervals (CI) by cross-classification of employment status and other measures of social disadvantage, 2001–2011, Belgian men and women aged 25–59 years.

	Men		Women	
	ASMR (95% CI)	MRR (95% CI)	ASMR (95% CI)	MRR (95% CI)
Employed—high educated	175.2 (171.8–178.7)	ref.	105.9 (102.6–109.2)	ref.
Employed—mid educated	243.0 (238.8–247.2)	1.41 (1.38–1.45)	126.2 (122.4–130.0)	1.23 (1.18–1.28)
Employed—low educated	304.3 (299.7–308.9)	1.72 (1.67–1.76)	138.6 (134.5–142.7)	1.28 (1.23–1.34)
Unemployed—high educated	434.5 (397.4–471.6)	2.34 (2.14–2.56)	180.1 (159.1–201.2)	1.73 (1.54–1.95)
Unemployed—mid educated	577.8 (541.9–613.8)	3.34 (3.13–3.57)	188.9 (174.2–203.5)	1.90 (1.75–2.06)
Unemployed—low educated	604.1 (578.8–629.5)	3.49 (3.33–3.67)	208.0 (196.4–219.6)	1.97 (1.85–2.10)
Employed—owner	218.7 (216.2–221.1)	ref.	110.8 (108.6–112.9)	ref.
Employed—tenant	349.9 (343.2–356.6)	1.62 (1.58–1.66)	165.6 (160.2–170.9)	1.49 (1.44–1.55)
Unemployed—owner	445.6 (423.4–467.7)	1.95 (1.85–2.05)	166.0 (156.9–175.1)	1.49 (1.41–1.59)
Unemployed—tenant	699.2 (669.4–728.9)	3.34 (3.19–3.49)	249.1 (232.3–265.8)	2.42 (2.27–2.59)
Employed—with partner	216.7 (214.3–219.2)	ref.	110.8 (108.6–113.0)	ref.
Employed—without partner	357.8 (351.0–364.6)	1.68 (1.64–1.72)	154.1 (149.5–158.7)	1.39 (1.34–1.44)
Unemployed—with partner	415.5 (394.7–436.3)	1.87 (1.77–1.97)	169.3 (160.2–178.5)	1.53 (1.45–1.63)
Unemployed—without partner	748.2 (716.4–780.0)	3.61 (3.45–3.77)	241.0 (224.9–257.1)	2.33 (2.18–2.50)
Employed—Native	245.3 (242.9–247.8)	ref.	122.4 (120.3–124.5)	ref.
Employed—Non-native	222.9 (215.8–230.0)	0.92 (0.89–0.96)	114.6 (107.8–121.5)	0.94 (0.88–1.00)
Unemployed—Native	650.1 (627.8–672.4)	2.59 (2.50–2.69)	208.1 (199.0–217.2)	1.72 (1.64–1.81)
Unemployed—Non-native	354.1 (327.7–380.5)	1.51 (1.40–1.63)	136.8 (120.5–153.2)	1.24 (1.11–1.39)

Mortality rate ratios adjusted for attained age. Ref. is reference category. Number of men = 1,693,799; number of women: 1,390,338.

For instance, high-educated employed men have an all-cause ASMR of 175.2 (95% CI: 171.8–178.7) whereas the ASMR for high-educated unemployed men was 434.5 (95% CI: 397.4–471.6) as compared to 604.1 (95% CI: 578.8–629.5) among low-educated unemployed men (Table 5). However, when we look at the cross-classification between unemployment and ownership, we observed a cumulative disadvantage effect on mortality. Men and women who were both unemployed and tenants of the home they lived in had a highly disadvantageous mortality pattern compared with people who were exposed to only one of the indicators of social disadvantage. Also, when it comes to the living situation, the results showed that unemployed men and women living without partner were more likely to die compared with employed men and women, as well as compared with unemployed men and women living with a partner. Men and women with a migrant background had a mortality advantage compared with native Belgians, but the effect of being unemployed seemed to be stronger than migrant background.

Additionally, we estimated the association between unemployment and all-cause and cause-specific mortality, adjusted for educational level, home ownership, living situation and migrant background (Table 4, model 2). Adding the other measures of social disadvantage to the model resulted in a significant decrease (the confidence intervals do not overlap) of the association between unemployment and all-cause mortality. This holds true for men and women, but the decrease was larger for men. Yet, a distinct mortality disadvantage for the unemployed people persisted for all COD, with dying from hypertensive diseases being the only exception. In the unadjusted model unemployed men and women had about twice as much chance to die from hypertensive diseases (MRR_men_: 1.86; 95% CI: 1.01–3.43 and MRR_women_: 2.37; 95% CI: 1.10–5.12) where in the adjusted model the effect was explained. For all other COD among women, adding the indicators of social disadvantage to the model did not result in a significant decrease of the association between unemployment and mortality. However, among men, adjusting for indicators of social disadvantage resulted quite often in a decrease of the association, which was the case for mortality due to cancer, mental disorders, circulatory diseases, respiratory diseases, digestive diseases and external COD.

## Discussion

### Methodological issues

This study precisely assessed the relation of unemployment and cause-specific mortality among the working age population in Belgium, both in absolute and relative terms. For doing so, we disposed of an exhaustive, high-quality database including the total de jure population in Belgium. Moreover, the data from the three different sources (Belgian National Registry, census, and death certificates) have been individually linked, thereby avoiding a numerator-denominator bias. The wealth of this individual-level dataset enabled us to adjust the association between unemployment and cause-specific mortality for other measures of social disadvantage. Unemployment is correlated with other sources of social disadvantage, such as reduced financial means or increased likelihood of divorce [14,20]. At the same time, persons without jobs might be more vulnerable than others, e.g. in terms of education, gender, age or family situation [9,16]. We can thus assume that the effect of unemployment on health might not be evenly distributed across the social strata, which need to be controlled for in the analyses [9,27,34].

Unemployment status was measured at baseline (census 2001), however we did not have information on the history of the employment trajectory, nor on the reasons for the job loss. Timing is an important indicator in this regard because the association between joblessness and health might be stronger with longer durations of unemployment [11,21]. Cumulative time spent in unemployment is associated with poor health [7], which is problematic as current employment trajectories are more and more fragmented and unstable. On the other hand, being unemployed as a result of an involuntary job loss or a mass lay-off may have a different association with health than being unemployed because of own resignation [15]. Additionally, we did not have information on health behaviours or health expenditure, which are thought to account (at least partly) for the higher mortality levels among the unemployed [22,26].

Finally, we should address the issue of health selection. The causal link between unemployment and poor health is likely to be bidirectional [1,9,13–17,21]. Since the aim of this study was to estimate the association between unemployment and mortality, it was essential to avoid a health selection effect. Although we cannot fully exclude the possibility that poor health leads to unemployment, we reduced this probability by excluding individuals older than 59 years, Belgians who did not report good or very good self-rated health at baseline (census 2001), as well as individuals who were retired, who never had a job, who were unemployed but not actively looking for a job, and who were unemployed due to familial, social, personal, or health reasons. Sensitivity analyses showed that the mortality rates were indeed higher among the ‘unhealthy group’. All-cause mortality rates among unhealthy employed and unemployed people were twice as high compared with their healthy counterparts. Hence, by excluding these unhealthy people at baseline, we assumed that among this subset of healthy individuals, unemployment was generally not caused by poor health [13], instead we assume that the elevated mortality rates among the unemployed are related to the joblessness itself. Moreover, the sensitivity analyses showed that even in the ‘unhealthy group’, employment status remains associated with lower all-cause and cause-specific mortality (with only few COD as exceptions). This suggest that being at is associated with better health, even among this ‘unhealthy group’.

### Theoretical considerations on the main findings

This study revealed a distinct all-cause and cause-specific mortality disadvantage for unemployed men and women compared with their employed counterparts. This finding is consistent with the literature on unemployment and health [7,9,11,12,17,20] and therefore not surprising. The mortality inequalities by employment status were rather high. We would have expected that in a context of a welfare state with a generous social security system ensuring unemployment pays and people’s access to care (as is the case in Belgium), that there would have been smaller mortality differences by employment status [20]. Part of the explanation however, could be the fact that in 2001 the unemployment rate in Belgium was rather low (6.6%) [35]. Previous research already proved that the excess mortality among the unemployed compared with the employed is larger in times of low national unemployment rates [15–17,23]. When the overall unemployment rate is low, health selection effects of getting unemployed may be stronger [6]. These health effects may operate both direct and indirectly: both people in poor health (direct) and people with higher risk factors for morbidity and mortality may be at a higher risk of getting or being unemployed. By selecting a healthy sample, we probably will have limited the direct health selection effect, but probably less the indirect health effect. As mentioned before, probably both mechanisms (causation and selection) will be at work.

The causal mechanisms is related to the rewards that are involved with paid work: financial security, social contacts, a sense of meaning, structured daily activities, as well as psychosocial rewards such as self-esteem or prestige [11,14]. Consequently, job loss has its negative consequences in several ways as well. The lack of financial security [11,12,15,21,22] might affect the use of health care and is also a continuous source of stress. Unemployed people are also more likely to have lower psychological wellbeing and higher levels of stress [12–14,20–22], which may eventually lead to a deterioration of physical health as well [20]. Moreover, a higher likelihood of risk behaviour, such as tobacco or alcohol consumption, has been observed among the unemployed [12,17,22,25].

The excess mortality among unemployed men and women was particularly elevated for the COD related to adverse health behaviours, especially to alcohol and tobacco consumption and external COD. This is in line with several studies that observed a correlation between being unemployed and problematic alcohol consumption [10,12], alcohol-related morbidity [12] or even mortality [9,12,21]. This relationship could be explained in two ways. Firstly, alcohol use could lead to unemployment; and secondly, alcohol use might be triggered by the stress involved with being unemployed [36]. Since we did not obtain data on health behaviours, we cannot be sure about the direction of the association. However, we assume that in our ‘healthy sample’ the explanation might be more likely to be found in the stress-related health behaviour hypothesis. This does not imply that we exclude the fact that the adverse health effects of unemployment could be more concentrated among the already more vulnerable people in terms of alcohol consumption [12]. The observation that excess mortality was mainly observed for the behavioural COD and less for the COD associated with health care use, can be explained by the organization of care in Belgium, which is highly accessible through the system of social security. Moreover, for some COD (e.g. cancer), the observation period of eleven years might be too short to observe inequalities.

Both unemployed men and women were at a higher risk of dying compared with their employed counterparts. However, the association was more pronounced among men, as was observed in other studies as well [7,10,13,19]. Largely, this difference can be attributed to the different traditional roles of men and women in society [7,13,14,19]. Men remain most often the main economic provider within the household, while women are more often involved in the role of caregiver and housekeeper for the household. Therefore, having a good job and financial stability may have more effect on men’s health than on women’s health. Unemployed women with a family often have their basic income needs guaranteed by their partner’s income. Additionally, opposed to men, when women lose their job, they are more likely to replace the rewards they would gain from the job, by alternative rewards gained from their nursing role in the household [13,19]. Following this idea, we assume that living without a partner and being unemployed might have a more detrimental effect on health for women, whereas for men mortality would be highest among the unemployed living with a partner [13,21]. Our findings however suggested that both men and women living without a partner have a higher mortality risk compared to their counterparts living with a partner (both employed and unemployed). A protective effect of living with a partner has thus been observed for women (as expected), but also for men. A possible explanation for this could be the tendency that men are more inclined to engage in unhealthy and risky behaviours, unless they live with a partner who exerts some kind of social control on them.

Furthermore, the results proved that unemployment was indeed associated with other measures of social disadvantage: low education, being tenant of a dwelling, living without partner and having a migrant background. On the other hand, notwithstanding having a high position on other social scales (e.g. being high-educated or being owner of a dwelling), being unemployed remained associated with increased all-cause and cause-specific mortality, although mortality was higher among the lower SE groups. Moreover, the results showed that the other measures of social disadvantage did only slightly decrease the elevated cause-specific mortality risks among the unemployed. Yet, the results suggested that employment status seemed to be a more important marker for one’s health than the other indicators of social disadvantage. The results also proved clearly that the association between unemployment and mortality not only varied by gender, but also by other measures of social disadvantage, especially the living situation and the material means (i.e. ownership), which is in line with previous research [13,21]. Thus, accounting for the social composition of the unemployed group is crucial when analysing health effects [9]. It seemed that the mortality risks were particularly high in the most vulnerable segments of the population, i.e. men and women living without a partner and tenants of dwellings. On the other hand, while some suggest that unemployment has a more detrimental health effect for high-educated men, because of their higher career expectations and the potentially larger losses [8], we observed a reversed gradient. Among the unemployed, low-educated men had higher mortality risks compared with high-educated men, whereas for women, mortality was equally across educational attainment.

## Conclusions

Following the higher unemployment levels among the lower social strata, especially in combination with simultaneously being in other lower social strata, implies for urgent matters [7]. Special and tailored attention should be paid to tackle the adverse health status of these vulnerable groups [13] such as people living without a partner or financially less secure groups. In this regard, ensuring financial security, good housing and accessible health care provision are key [10].

We observed a strong association between unemployment and mortality. Yet, we cannot make conclusion on the causal directions. To enhance our knowledge, future research should repeat this kind of analyses in contexts with high unemployment levels [17]. This study was based on a sample of healthy people, but without information on the employment history, which future analyses should take into account. Moreover, we focused on mortality, which is of course the extreme end of health problems. Future studies should also analyse the association between unemployment and morbidity, while considering gender differences and the social context. Next to being unemployed, attention should also be paid to other employment regimes such as part-time work, precarious work, temporal employment and job insecurity, which may be also harmful to health [11]. Finally, it would be interesting to probe into the health effect of unemployment on the relatives of unemployed men and women as well.
